# Structural Analysis and Adsorption Studies of (PbO, MgO) Metal Oxide Nanocomposites for Efficient Methylene Blue Dye Removal from Water

**DOI:** 10.3390/ma17122890

**Published:** 2024-06-13

**Authors:** Saloua Helali, Mohamed Rashad, Anouar Ben Mabrouk, Munirah A. A. Alanazi, Manahil S. Mustafa

**Affiliations:** 1 Department of Physics, Faculty of Sciences, University of Tabuk, King Faisal Road, Tabuk 47512, Saudi Arabia; m.ahmad@ut.edu.sa (M.R.); ma.alanazi@ut.edu.sa (M.A.A.A.); 2 Department of Mathematics, Faculty of Sciences, University of Tabuk, King Faisal Road, Tabuk 47512, Saudi Arabia; amabrouk@ut.edu.sa; 3 Department of Statistics, Faculty of Sciences, University of Tabuk, King Faisal Road, Tabuk 47512, Saudi Arabia; msida@ut.edu.sa

**Keywords:** MgO, PbO, nanoparticles, XRD, HRTEM, FTIR, optical properties, adsorptions studies

## Abstract

In the present work, magnesium oxide (MgO) and lead oxide (PbO) nanoparticles were prepared by the co-precipitation method. Their structural parameters and morphology were investigated using XRD, HRTEM, and FTIR. The formation of the phases was seen to have small average crystallite sizes and an orthorhombic crystal structure for both MgO and PbO nanoparticles. The results of HR-TEM showed irregularly shaped nanoparticles: quasi-spherical or rod-like shapes and spherical-like shapes for MgO and PbO nanoparticles, respectively. The produced nanoparticles’ size using X-ray diffraction analysis was found to be 17 nm and 41 nm for MgO and PbO nanoparticles, respectively. On the other hand, it was observed from the calculations that the optical band gap obeys an indirect allowed transition. The calculated values of the band gap were 4.52 and 4.28 eV for MgO and PbO NPs, respectively. The MB was extracted from the wastewater using the prepared composites via absorption. Using a variety of kinetic models, the adsorptions were examined. Out of all the particles, it was discovered that the composites were best. Furthermore, of the models currently under consideration, the pseudo-second-order model best fit the degradation mechanism. The resultant composites could be beneficial for degrading specific organic dyes for water purification, as well as applications needing a wider optical band gap.

## 1. Introduction

The textile, paper, leather, and cosmetic industries, among others, significantly contribute to the vibrant and diverse array of colors in consumer products through the use of dyes. While these dyes enhance the visual appeal of various materials, their persistence and resistance to degradation make them particularly problematic when released into natural water bodies. Additionally, the sheer volume and variety of dyes used in industries demand innovative and sustainable solutions to mitigate their impact on the environment. As the global demand for clean water intensifies, innovative and efficient wastewater treatment methods become essential [1,2]. Wastewater has been treated using a variety of methods to remove colors, including adsorption [3,4], advanced oxidation [5], biological treatment [6], nanofiltration [7], and electrocatalysis [8]. Among these methods, adsorption stands out as a versatile and effective approach for removing diverse pollutants from wastewater. Adsorption is a physicochemical process where molecules or particles from a fluid adhere to the surface of a solid material, known as an adsorbent.

In the context of wastewater treatment, adsorption plays a crucial role in capturing and removing contaminants, such as heavy metals, organic compounds, and dyes, from the water matrix. The adsorption method relies on the principle that certain materials, due to their specific surface properties, have a strong affinity for particular pollutants, facilitating their selective removal. Various adsorbents, both natural and synthetic, are employed in wastewater treatment processes. Commonly used adsorbents include metal oxides [9,10], MXenes [11], chitosan [12], carbon-based nanostructures [13], and nanocomposites (NCs) [14]. Nowadays, nanotechnology has emerged as a field for addressing the ever-growing challenges of wastewater treatment. Nanocomposite adsorbents offer unprecedented opportunities to enhance the efficiency and selectivity of adsorption processes in removing contaminants from wastewater.

Many researchers are currently attracted by the use of nanomaterials in their oxide forms in environmental waste and toxics management, enhanced ceramics production, and catalysis. Metal oxide nanoparticles exhibit distinct chemical, physical, and electronic properties compared to their bulk counterparts. Their high surface-area-to-volume ratio, tunable morphology, and exceptional reactivity make them ideal candidates for numerous applications spanning from catalysis and sensing to medicine and environmental remediation. Numerous selective metal oxide nanoparticle adsorbents have been recognized as possessing the ability to recover organic dyes selectively. However, lead oxide has several interesting physics aspects, including its optical, electrical, and thermal properties, as well as its role in various industrial and environmental processes. PbO exists in two distinct crystalline forms: massicot (orthorhombic crystalline structure) and litharge (tetragonal crystalline structure) [15,16]. A variety of processes were used to create PbO nanostructures, including chemical deposition [17], sol–gel pyrolysis [18], thermal breakdown [19], and decomposition under microwave radiation [20]. Meanwhile, magnesium oxide (MgO) has exceptional surface properties at the nanoscale, due to its polyhedral structure and the formation of Frenkel or Schottky defects at the edge or corner [21,22,23]. These characteristics provide the nanomaterials with a large surface area, which makes them useful for cleaning up the environment. Its high melting point, excellent thermal conductivity, and remarkable electrical properties make it invaluable in manufacturing processes. Several techniques, such as sol–gel [24], chemical perception [25] and vapor-phase oxidation [26], have been used to synthesize MgO nanomaterials.

Therefore, this study aims to demonstrate the nanopowder properties of PbO and MgO produced through the co-precipitation method. The PbO and MgO nanoparticles were carefully analyzed utilizing a variety of analytical techniques, including high-resolution transmission electron microscopy (HRTEM), X-ray diffraction (XRD), and Fourier transformation infrared (FTIR). An optical analysis of the MgO and PbO NPs was conducted. Additionally, different kinetics models were examined along with their adsorption performance for different shaking times to eliminate the methylene blues from the wastewater.

## 2. Materials and Methods

### 2.1. Materials and Reagents

MgO and PbO NPs were prepared using the co-precipitation method using magnesium nitrate or lead nitrate and sodium hydroxide without additional purification, which was reported previously by the NanoFab technology Company, Cairo, Egypt [27]. The purity of the starting materials was 99.98%. An aqueous solution of 0.1 M of lead and magnesium nitrates was stirred continuously for an hour using a magnetic stirrer to completely dissolve the lead and silver nitrates. Additionally, a 0.8 M aqueous solution of sodium hydroxide (NaOH) was prepared in the same manner. After the complete dissolution of the lead nitrate, 0.8 M NaOH aqueous solution was added dropwise for 45 min. After the reaction was finished, the solution was allowed to settle for an entire night before the supernatant was carefully removed.

### 2.2. Sample Characterizations

The Shimadzu XD-3A (Kyoto, Japan) X-ray diffractometer was used to assess the crystallinity of the as-prepared MgO and PbO NP films. Using a monochromatic Cu Kα source (where λ = 1.54 Å), the XRD spectra were collected from 4° to 80° with a step size of 0.06°.

The produced PbO and MgO nanoparticles were analyzed using high-resolution transmission electron microscopy (HRTEM) to find out more about their structural shape and particle size distribution. The JEOL JEM 2100 model plus from Tokyo, Japan, was used to analyze the morphology. A Perkin Elmer (Waltham, MA, USA) lambda 750S UV-vis spectrophotometer was used to obtain UV-vis absorbance spectra.

A Thermo-Nicolet-6700 FTIR spectroscope (Waltham, MA, USA) was utilized for measuring the FTIR spectra for the composites in the wavenumber window of 4000–400 cm^−1^.

A LAMBDATM 750 UV/Vis/NIR spectrophotometer (National, CA, USA) operating in the 200–800 nm wavelength range was used to measure the absorbance at room temperature.

### 2.3. Adsorption Experiment

The batch adsorption measurements were conducted at 5 mg/L using methylene blue solution by introducing predetermined dosages of PbO and MgO NPs in 100 mL Erlenmeyer flasks. The flasks were placed in front of a Lab Tech shaking instrument model that was shaken at room temperature and 150 rpm. The flasks were taken out every ten minutes, and a UV-vis spectrophotometer calibrated to 660 nm was used to determine the amount of unadsorbed methylene blue. Equation (1) was used to compute the adsorption efficiency [28]:

(1) $$efficiency\;(\%)=\frac{C_{o}-C_{t}}{C_{o}}\times 100=\frac{A_{o}-A_{t}}{A_{o}}\times 100$$

where *C_o_* and *C_t_* are initial and residual MB concentrations, respectively. *A_o_* and *A_t_* are the initial and residual MB absorbance, respectively.

## 3. Results and Discussion

### 3.1. Characterizations of PbO and MgO Nanoparticles

**Figure 1:**
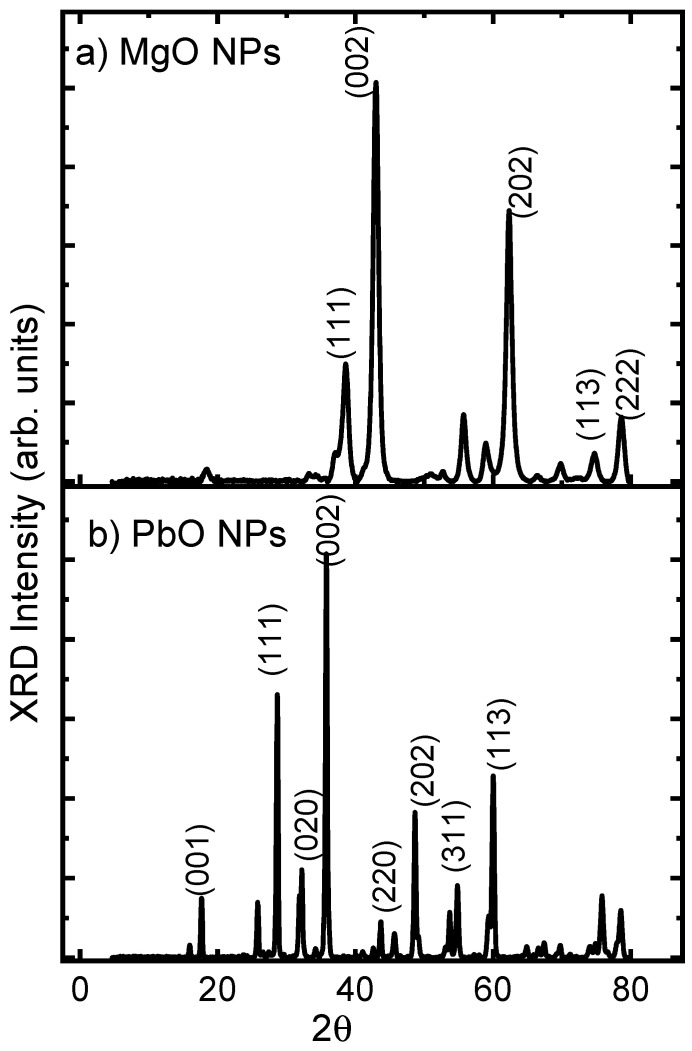
XRD of (**a**) PbO and (**b**) MgO NPs.

Figure 1 displays the XRD pattern of the powder of MgO and PbO NPs. The XRD patterns’ diffraction peaks only showed the phases of MgO and PbO NPs. The detected peaks [29] in Figure 1a at 18.57°, 36.96°, 38.02°, 42.98°, 62.36°, 74.71°, and 78.66° correspond to the (111), (002), (202), (113), and (222) planes (JCPDS No. 87-0653), indicating the creation of the polycrystalline cubic structure of MgO NPs. On the other hand, the XRD pattern of the powder of PbO nanoparticles is shown in Figure 1b. The prominent peaks, designated as the (001), (111), (020), and (002) planes, respectively, at 17.64, 28.62, 31.82, and 35.72 degrees, are typical peaks for the pure tetragonal phase of α-PbO NPs [26]. The purity of the nanomaterial was demonstrated by the absence of any additional peaks in this study.

The Debye–Scherrer equation (Equation (2)) was utilized to determine the size of the crystals. The equation involves four constants, the X-ray wavelength (λ = 1.5418 Å), the full-width at half-maximum intensity (β), the Braggs angle (θ), and the crystalline form constant, represented by

D = 0.9 λ/βcosθ(2)

According to this equation, the average crystallite sizes of PbO and MgO NPs are 41 nm and 17 nm, respectively.

**Figure 2:**
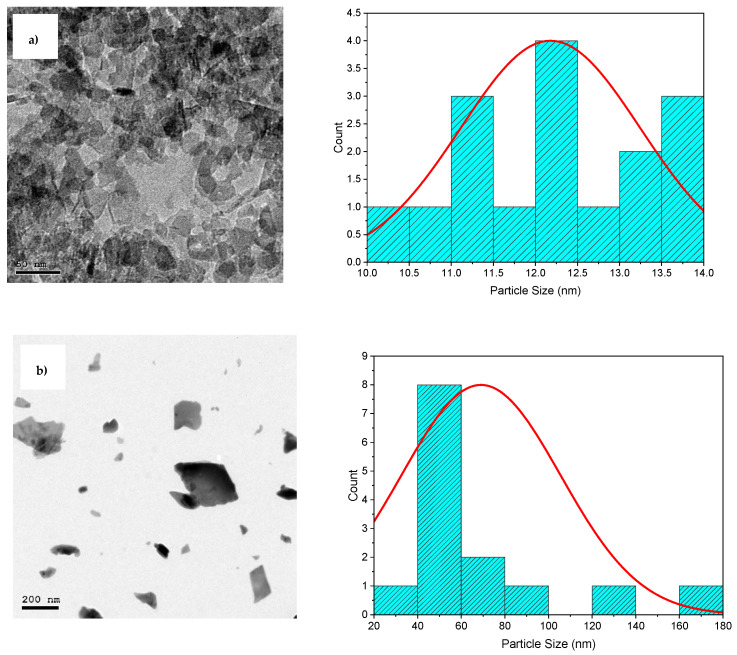
HRTEM images and particle size distribution of (**a**) MgO NPs and (**b**) PbO NPs.

HR-TEM analysis was carried out to obtain more information on the morphological characteristics of the MgO and PbO NPs. The results are shown in Figure 2. The figure depicts HR-TEM images with particle size distribution histograms. All the samples showed irregularly shaped nanoparticles: quasi-spherical or rod-like shapes and spherical-like shapes for MgO and PbO NPs, respectively. Moreover, the average particle sizes using the “Image J” (https://imagej.net/ij/) program were found to be 12.25 nm and 69 nm for MgO and PbO NPs, respectively. Since the estimated particle size from TEM pictures was more than the estimated crystallite size from XRD, it may be concluded that each particle was made up of many crystallites.

**Figure 3:**
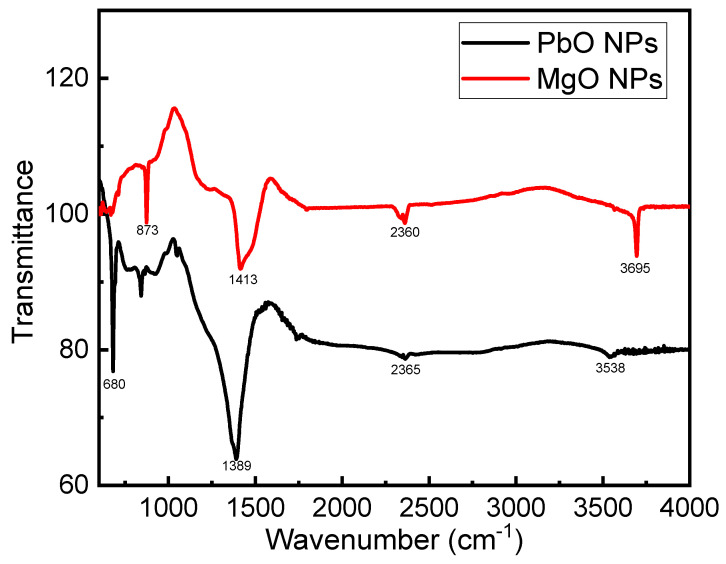
FTIR analysis of MgO and PbO NPs.

The PbO NP and MgO NP FTIR spectra’s 4000–400 cm^−1^ wavenumber region is displayed in Figure 3. The Pb-O bond stretching band is responsible for the distinctive transmittance peaks that are detected within the 680 cm^−1^ range. Oxides are also visible in this range due to decreased transmittance [30,31]. The stretching vibrations caused by the C-O bond are represented by an intense infrared absorption seen in the range of 2353. It is thought that during the PbO nanoparticle preparation process from Pb(CH_3_COO)_2_, some C-O groups were combined with the particles. Similar to this, an absorption band in the 3500–3600 range indicates the existence of OH, which is created during the nanoparticle synthesis process [32,33]. As seen in Figure 3, an FTIR analysis of MgO nanoparticles was performed. The existence of nano-sized MgO is verified by the appearance of significant peaks at 680 cm^−1^ [27] The (O–H) stretching mode of hydroxyl groups, which were present on the surface due to the moisture, accounts for the strong band that was observed at 3695 cm^−1^. However, the peak detected at 1421 cm^−1^ was traced back to the water molecules’ bending vibration [34].

### 3.2. Optical Investigations

**Figure 4:**
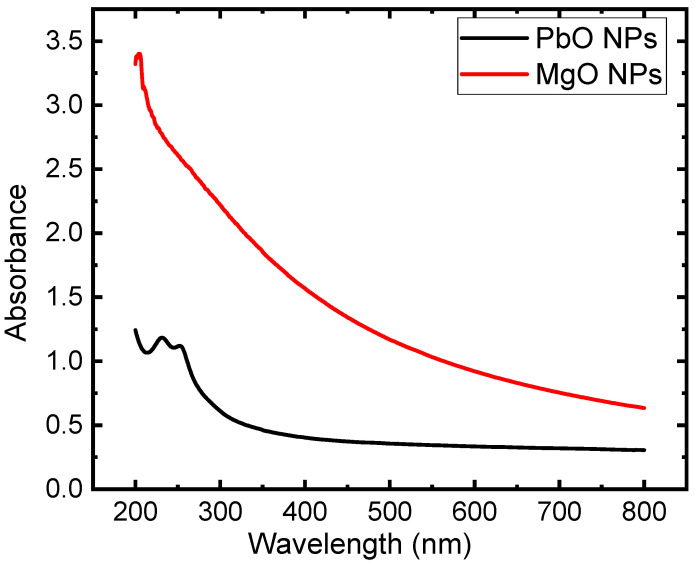
Absorbance spectra of PbO and MgO NPs.

The absorbance spectra exhibit an attractive trend in the 200–800 nm wavelength range, as demonstrated in Figure 4. For all spectra of MgO and PbO nanoparticles, in a wavelength range of 100–400 nm in the UVC region, all spectra show a maximum value for both nanoparticles. On the other hand, a plateau zone is shown in the wavelength range of 400–800 nm.

The following equation can be used to investigate the absorption coefficient [35]:

(3) $$\alpha =\frac{absorbance}{d}$$

where *d* is the sample thickness.

**Figure 5:**
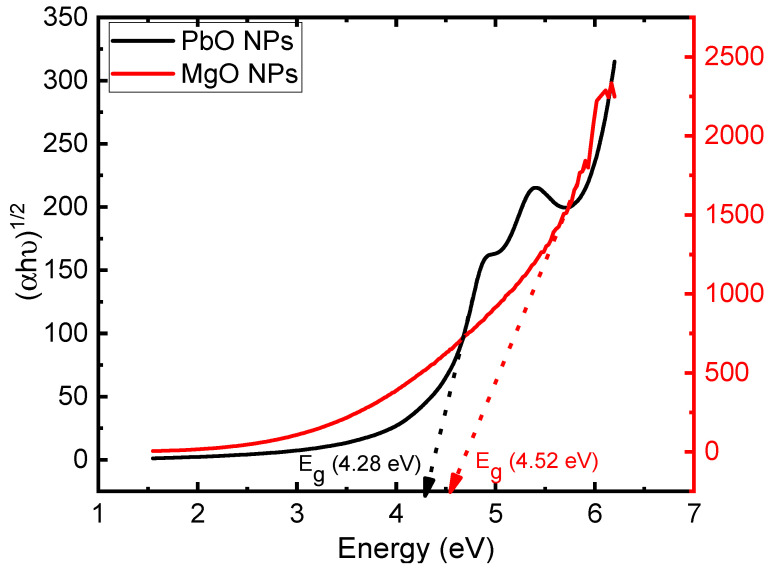
(αhν)^1/2^ versus hν of PbO and MgO NPs.

The equation that follows could be used to obtain the optical band gap:

(4) $${\left(\alpha h\nu \right)}^{n}=A(h\nu -E_{g})$$

where *A* is a constant, *E_g_* is the optical band gap, *hν* represents the energy of the incident spectrum, and *α* is the absorption coefficient. Furthermore, the transition process is described by *n*. When an indirect transition is permitted, the value of n is equal to 1/2. Conversely, if *n* is equal to 2, it is associated with the permissible direct transition. Figure 5 shows the relation between (*αhν*)^1/2^ and hν for MgO and PbO NPs. As a result, for MgO and PbO nanoparticles, the dominant transition process is the direct permitted transition. The calculated values of the band gap are 4.52 and 4.28 eV for MgO and PbO nanoparticles, respectively.

### 3.3. Methylene Blue Dye Adsorption Performance

**Figure 6:**
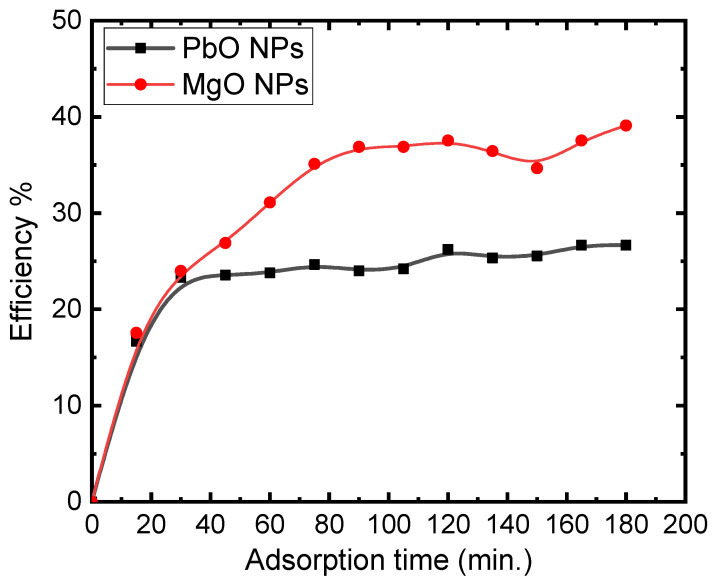
Adsorption efficiency of 5 mg/L of MB on the PbO and MgO NPs.

One of the most crucial factors for the effective usage of the adsorbent in a practical application is the contact time. The influence of contact time (0–180 min) on the adsorption efficiency of 5 mg/L of MB onto PbO and MgO NPs is presented in Figure 6. The results show a rapid adsorption rate during the first 20 min and reach equilibrium after roughly 40 min for the PbO nanocomposite and 90 min MgO NPs, respectively. In the first 30 min, over 25% of the MB on the NP samples was eliminated. This can be explained by more active sites that are initially available on the adsorbent for the adsorption of MB dye molecules. But as the process goes on, these active sites fill up and the dye molecules eventually saturate the adsorbent’s surface. The adsorbent becomes saturated after a certain amount of time, and the concentration gradient starts to stabilize. The results of this study led to the conclusion that 30 min is the ideal amount of time for the adsorption process and MgO NPs have a 10% better removal efficiency than PbO NPs, rising from 25% to 40% in 140 min.

The mass of methyl blue that was degraded per unit of NPs (adsorption capacity) (q, mg/g) at a fixed time and at the equilibrium state was measured [36]:

(5) $$q_{t}=(C_{i}-C_{t})\frac{V}{W}$$

(6) $$q_{e}=(C_{i}-C_{e})\frac{V}{W}$$

where *q_t_* is the quantity of degraded MB capacity at time (*t*), mg/g; *q_e_* is the quantity of degraded MB at the equilibrium state, mg/g; *C_i_* is the initial concentration of MB solution, mg/L; *C_t_* is the concentration of degraded MB at time (*t*), mg/L; *C_e_* is the equilibrium concentration of degraded MB, mg/L; *V* is the solution volume, *L*; and *W* is the mass, *g*.

**Figure 7:**
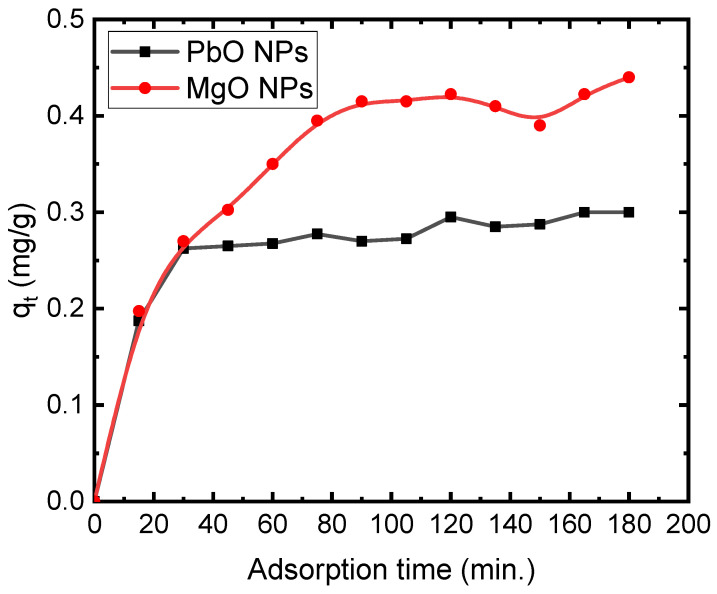
Adsorption capacity of 5 mg/L of MB on the PbO and MgO NPs.

The degradation dye capacity vs. shaking time for concentrations of 5 mg/L of MB is shown in Figure 7. As seen in the figure, the degradation capacity increases with time. The adsorbed MB value increases gradually with increasing contact time, reaches equilibrium within 30 min for PbO NPs, and reaches equilibrium within 70 min for MgO NPs. These data show that the rate of adsorption increases initially and then approaches a constant value as the contact time increases. This is because dye molecules tend to aggregate as contact times increase, making deeper diffusion into the adsorbent structure at higher-energy sites nearly impossible. The process of aggregation eliminates the impact of contact time, as the pores become fully occupied and provide resistance against the diffusion of aggregated dye molecules within the adsorbent materials. It has been observed that the one-hour equilibration time is adequate because maximum adsorption is reached during this time [37]. It is clear, from HRTEM, that the distribution of the particles in MgO NPs is more orderly. However, the particles are denser; therefore, the possibility of the adsorption capacity in the case of MgO NPs is more than in the case of PbO NPs. This reason gives MgO a greater MB removal effectiveness in comparison with PbO over time.

### 3.4. Adsorption Kinetic Studies

Researchers studied the kinetics to describe the dynamics of the adsorption process and identify the rate constant’s order [38]. Several models were used to evaluate the kinetics of MB adsorption on the surface of PbO and MgO nanoparticles, including the pseudo-first-order, pseudo-second-order, intraparticle diffusion model, and Elovich. Table 1 summarizes the different parameters, which were calculated based on the plots of the kinetic model equations, with correlation coefficients (R^2^).

**Figure 8:**
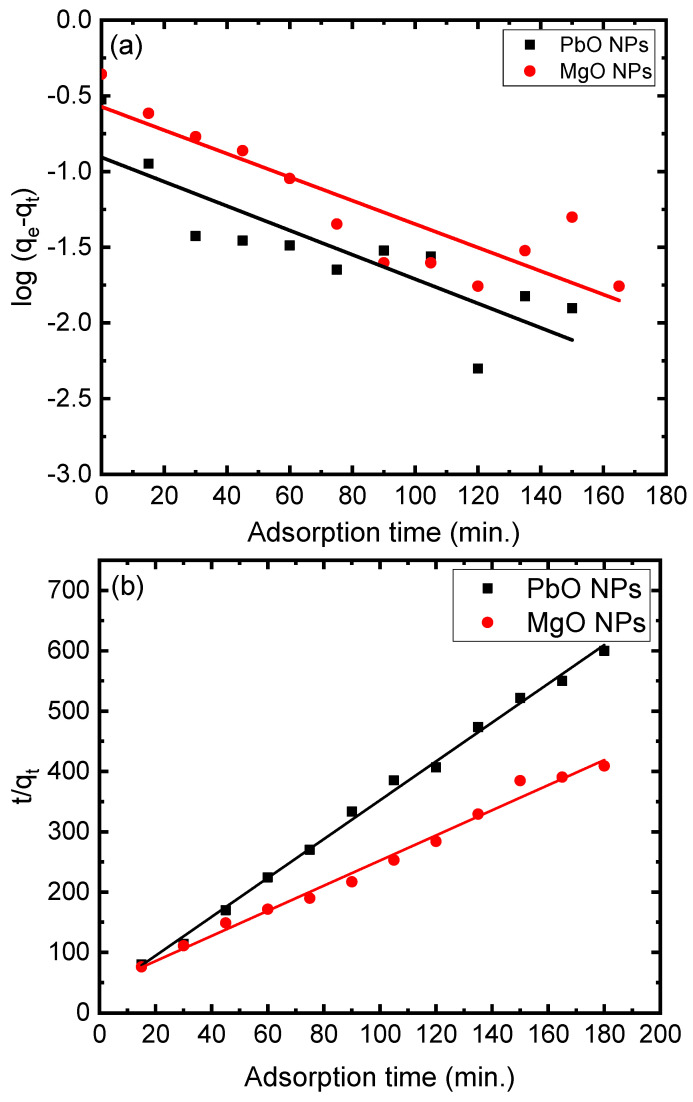

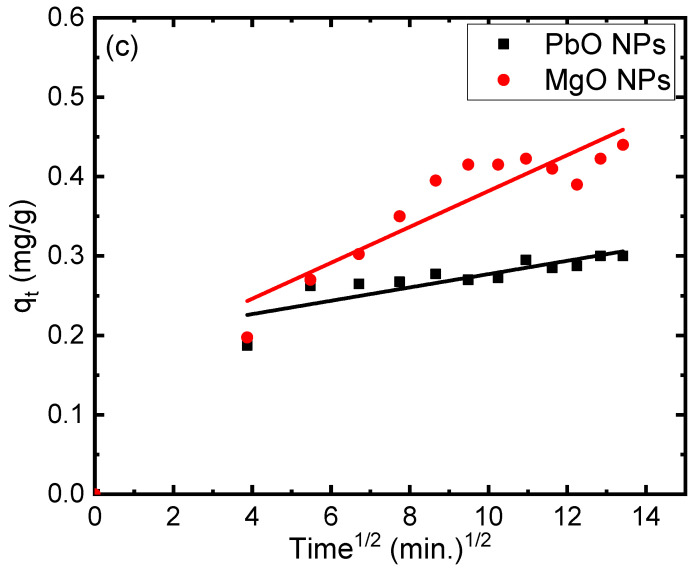
Linear fittings with (**a**) pseudo-first-order kinetics model, (**b**) pseudo-second-order kinetics model, and (**c**) intraparticle diffusion model of 5 mg/L of MB on the PbO and MgO NPs.

The graph of the three models versus shaking times is presented in Figure 8. First-order rate constants for the sorption of MB onto the PbO NP surface were estimated to be 0.019 L/min with a regression coefficient of 0.73, whereas it was 0.018 L/min with a regression coefficient of 0.78 for the MgO NPs. The regression coefficient values indicated that the pseudo-first-order kinetic model did not match well with the sorption data. The kinetic data were also examined using a pseudo-second-order kinetic model. Based on the kinetic parameters in Table 1, the pseudo-second-order kinetic model was found to be appropriate with a great correlation coefficient of 0.99. In order to examine the sorption data and look into the rate-limiting phase that may contribute to the prediction of the sorption mechanism, a variety of kinetic diffusion models were employed. For the PbO nanocomposite, the intraparticle diffusion graph behaved like a point that was 0.193 distant from the origin, while the MgO nanoparticle point was 0.156 with regression coefficients of 0.72 and 0.83, respectively. The intercept and regression values for the PbO and MgO nanoparticles showed that the thickness of the boundary layer around the solid particles may influence the sorption rate, indicating that intraparticle diffusion cannot be the only factor affecting the rate in both cases.

The Elovic equation is represented by the expression below [27]:

(7) $$q_{t}=\frac{\ln\left(\alpha \beta \right)+ln(t)}{\beta }$$

**Figure 9:**
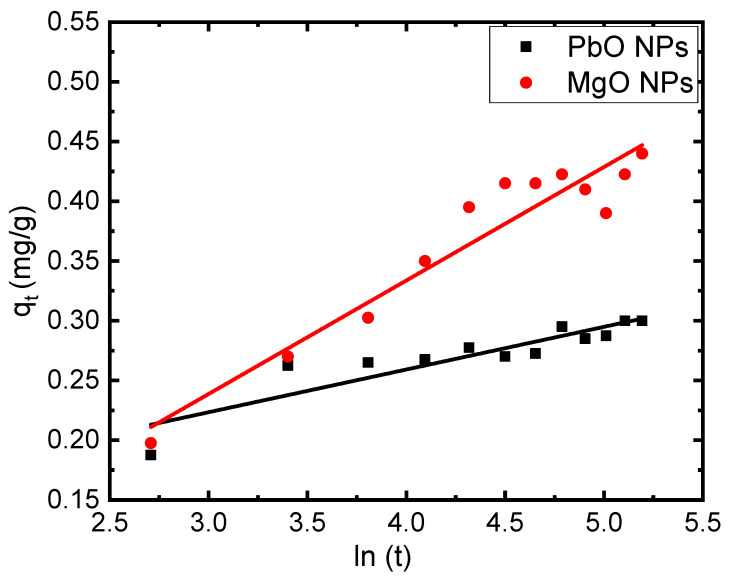
Plots of qt versus ln(t) versus time (t) of 5 mg/L of MB on the PbO and MgO NPs.

Another suggested equation for examining the adsorption of the studied sample is the Elovic equation. It applies to the examination of solutes that are adsorbing from a liquid solution. Figure 9 illustrates the relationship between qt and ln(*t*). The calculated values of sorption (*α*) and desorption (*β*) for the adsorption of MB in the presence of PbO and MgO NPs are 0.92 mg/g min and 28.01 g/mg, and 0.15 mg/g min and 10.53 g/mg, respectively.

Utilizing the Boyd kinetic equation, the experimental results were analyzed as the intraparticle diffusion plot’s dual characteristic suggests the participation of both intraparticle diffusion and surface sorption [28]. Boyd et al. suggested studying the diffusion mechanism throughout the adsorption process using the following mathematically stated model:

(8) $$B_{t}=-0.4977-ln(1-\frac{q_{t}}{q_{e}})$$

**Figure 10:**
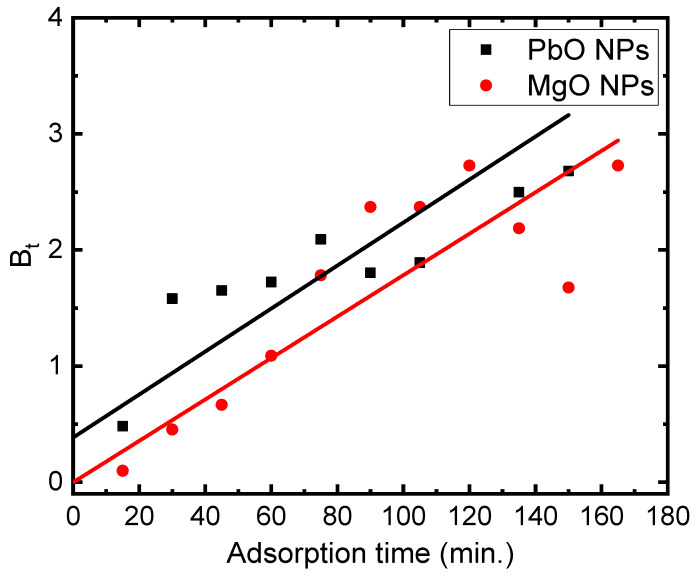
Plots of B_t_ versus the time (t) of 5 mg/L of MB on the PbO and MgO NPs.

Equation (8) is a mathematical function of (*q_t_*/*q_e_*), and this ratio provides the adsorbate adsorbed fraction at different shaker times. Graphs of *B_t_* against MB time (*t*) in the presence of PbO and MgO NPs are illustrated in Figure 10. The Boyd model asserts that the linear graph of *B_t_* versus time will cross the origin. This suggests that process control is in place for the particle diffusion mechanism. Conversely, one could consider diffusion to be the process’s rate-limiting step. The MB adsorption parameters in the presence of PbO and MgO NPs are illustrated in Table 1.

**Figure 11:**
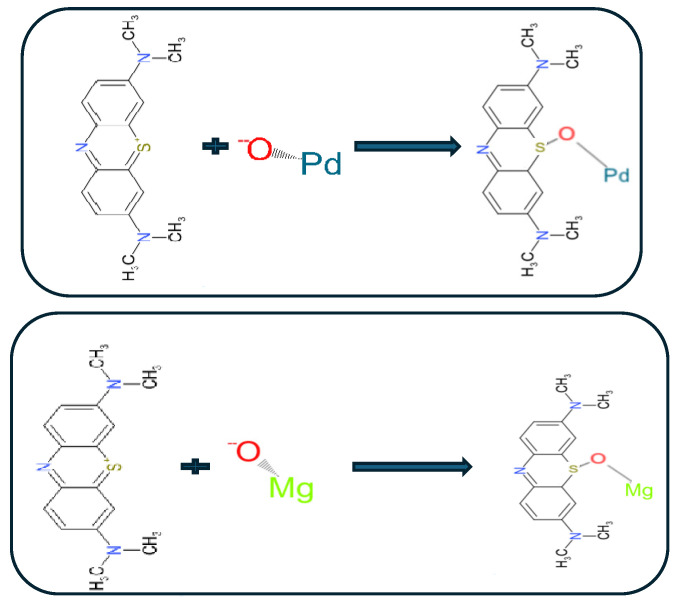
Mechanism of adsorption of MB dye onto PbO and MgO NPs.

The chemical reaction between adsorbate and adsorbents involves the exchange or sharing of electrons, leading to the formation of new chemical species or the alteration of existing ones. The adsorption process occurs through electrostatic attractions between the positively charged methylene blue molecules and the negatively charged surface of the lead oxide nanoparticles. The presence of oxygen-containing functional groups on the surface of lead oxide nanoparticles also facilitates chemisorption through Lewis acid–base interactions [39]. Accordingly, the following chemical reaction (Figure 11) can be used to represent the adsorption mechanism of MB by PbO and MgO NPs:

### 3.5. Comparison of Performance with Reported Oxides NPs

Adsorption studies on metal oxide nanocomposites for the removal of dyes have yielded valuable data contributing to the understanding of their efficacy in wastewater treatment. The collected data indicate that various metal oxide nanocomposites exhibit significant adsorption capacities for dyes commonly found in industrial wastewater. Furthermore, understanding the mechanisms underlying dye adsorption onto metal oxide nanocomposites is crucial for the design and optimization of nanocomposite materials tailored for efficient dye removal applications.

The current study’s kinetic studies were compared with those of previous metal oxide nanocomposite investigations concerning contact time and adsorption capacity. As Table 2 summarizes, our study has resulted in quick adsorption with a short contact time for MB adsorption, in contrast to the other preceding investigations. The choice of a lower beginning concentration range for the adsorption investigation is primarily to blame for the adsorption capacity attained in this work being lower than that in the reported work. Overall, the data collected from adsorption studies underscore the potential of metal oxide nanocomposites as effective adsorbents for dye removal in wastewater treatment.

## 4. Conclusions

Using the co-precipitation method, lead oxide (PbO) and magnesium oxide (MgO) nanoparticles were created. The structural parameters, optical band gap (Eg), and their adsorption of methylene blue were studied using different models. Quasi-spherical or rod-like shapes and spherical-like shapes were determined for MgO and PbO NPs, respectively. Moreover, the average particle sizes were 12.25 nm and 69 nm for MgO and PbO NPs, respectively. A strong peak in the MgO nanoparticles can be noticed by FTIR analysis at 873 cm^−1^ and verifies the presence of peroxo groups in the breadth of the MgO vibration at 3693 cm^−1^. The PbO nanoparticle’s 680 cm^−1^ broadband peaks were produced. The optical band gap was determined at 4.52 and 4.28 eV for MgO and PbO nanoparticles and it obeys the indirect allowed transition. The prepared nanoparticles show a good catalytic performance in removing the MB from the wastewater. The pseudo-second-order model is the best model for describing the degradation mechanism among the investigated models. The prepared MgO and PbO NPs could qualify for the degradation of some organic dyes for water purification and could be used for other applications.

## Figures and Tables

**Table 1:** Methylene blue adsorption parameters in the presence of PbO and MgO NPs.

Model	Parameter	Adsorbent	
		PbO	MgO
Pseudo-first-order kinetic model $\log\left(q_{e}-q_{t}\right)=logq_{e}-K_{1}\frac{t}{2.303}$	*q_e_*_,*exp*_ (mg/g)	0.3	0.4
	*q_e_*_,*cal*_ (mg/g)	7.96	3.73
	[*q_e_*_(*exp*)_ − *q_e_*_(*cal*)_] (mg/g)	7.66	3.33
	*K*_1_ (min^−1^)	0.019	0.018
	R^2^	0.73	0.78
Pseudo-second-order kinetic model $\frac{t}{q_{t}}=\frac{1}{K_{2}{q_{e}}^{2}}+\frac{t}{q_{e}}$	*q_e_*_,*cal*_ (mg/g)	0.311	0.481
	[*q_e_*_(*exp*)_ − *q_e_*_(*cal*)_] (mg/g)	0.011	0.081
	*K*_2_ × 10^−4^ (g/mg·min)	0.343	0.099
	R^2^	0.99	0.99
Intraparticle diffusion kinetic model $q_{t}=K_{diff}\times {\left(t\right)}^{1/2}+C$	*K_diff_* (mg/g·min^1/2^)	0.008	0.022
	*C*	0.193	0.156
	R^2^	0.72	0.83
Elovic kinetic model $q_{t}=\frac{\ln\left(\alpha \beta \right)+ln(t)}{\beta }$	*α* (mg/g·min)	0.92	0.15
	*β* (g/mg)	28.01	10.53
	R^2^	0.82	0.92

*q_e_* (mg/g): adsorption capacity; *K*_1_ (min^−1^): rate constant of pseudo-first-order model; R^2^: R-squared value; *K*_2_ (g/mg·min): rate constant of pseudo-second-order model; *C*: intercept; *K_diff_* (mg/g·min^1/2^): rate constant of Temkin model; *α* (mg/g·min): sorption rate; *β* (g/mg): desorption constant.

**Table 2:** Comparison of adsorption capacity of PbO NPs and MgO NPs employed as adsorbents in this study with other metallic oxide NPs concerning MB.

Adsorbent	Adsorption Capacities (mg/g)	Contact Time min	References
NiO	8.42	500	[39]
CuO	3.62	500	
Fe_2_O_3_ NPs	0.56	-	[40]
F+-MgO NPs	0	105	[41]
Gd_0.5_Sr_0.5_FeO_3_ NPs	1.45	30	[42]
ZnO Thin Films	6.5	50	[43]
TiO_2_ Thin Films	10.5	50	
MgO NPs	0.481	30	This work
PbO NPs	0.311	30	This work

## Data Availability

Data are contained within the article.
